# The complete mitochondrial genome of a tertiary relict evergreen woody plant *Ammopiptanthus mongolicus*

**DOI:** 10.1080/23802359.2017.1413301

**Published:** 2017-12-12

**Authors:** Tingqiao Yu, Lichun Sun, Hongwei Cui, Shengli Liu, Jingyu Men, Shaoliang Chen, Yuzhen Chen, Cunfu Lu

**Affiliations:** Beijing Advanced Innovation Center for Tree Breeding by Molecular Design, College of Biological Sciences and Biotechnology, Beijing Forestry University, Beijing, China

**Keywords:** *Ammopiptanthus mongolicus*, mitochondrial genome, phylogeny

## Abstract

*Ammopiptanthus mongolicus* is a tertiary relict evergreen broad-leaf shrub in family Fabaceae with remarkable tolerance to desiccation and low temperature. In this study, we report the complete mitochondrial genome of *A. mongolicus.* The total genome length was 475,396 bp and contained a total of 127 genes, including 79 protein-coding genes (28 novel genes, 45 known functional genes, and six known orf genes), three rRNA genes, and 45 tRNA genes. Most of the genes were single-copy genes, only six were duplicated and two were multi-copy. The mitochondrial genome also contained ‘promiscuous’ sequences from the chloroplast, 16 intact tRNAs of mitochondrial origin, and 29 intact and potentially functional chloroplast-derived tRNAs. The overall GC content of the mitochondrial DNA was 42.75%. A neighbour-joining phylogenomic analysis showed that *A. mongolicus* was closely related to *Medicago truncatula*, which also belongs to family Leguminosae.

*Ammopiptanthus mongolicus* is an evergreen shrub in Leguminosae, which is believed to have originated from the Tertiary period (from 65 to 2 million years ago) (Liu et al. 1995). Increasing desertification in central Asia and anthropogenic activities (e.g. coal mining, road construction, overgrazing and cutting for firewood) are causing continual habitat losses and reductions in the populations of *A. mongolicus*, to such an extent that the species has been categorized as ‘endangered’ and given protected status in China (Fu and Jin 1992). This species primarily inhabits the desert of northwestern China where the annual precipitation is often less than 50–100 mm, and the temperature ranges from −30 °C in winter to 40 °C in summer (Liu et al. 2013). Given its ancient origin and survival in a persistently arid environment, *A. mongolicus* can withstand drought and low-temperature conditions through morphological and physiological adaptations (Cao et al. 2009; Shi et al. 2016). Plant mitochondria play specific roles during exposure to harsh environments (Jacoby et al. 2012), so it is important to sequence and annotate the mitochondrial genome (mito-genome) of *A. mongolicus*. Here, we report the complete mito-genome sequence of *A. mongolicus* by next-generation sequencing. The annotated mitochondrial DNA (mtDNA) sequence has been deposited in GenBank under accession no. MK 683210.

In this study, *A. mongolicus* seeds collected from Bayannaoer in Inner-Mongolia of China (105°12'–109°53′E, 40°13'–42°28′N) were germinated and grown on hormone free MS medium in dark. The mtDNA was extracted from 100 grams of fresh young yellow aseptic *A. mongolicus* seedlings. Separation of *A. mongolicus* mtDNA was performed using a modified sucrose gradient ultracentrifugation method according to Zsigmond et al. (2008). The extracted mtDNA was sequenced by BGI (Shenzhen, China) on an Illumina HiSeq 4000 platform (Illumina, San Diego, CA) from a 500-bp paired-end library, which generated 1637 Mb raw data and 13 million 125-bp reads and deposited in the herbarium of the Beijing Forestry University (Beijing, China).

To decrease the redundant data, filtration were conducted on the raw data. The clean data were assembled using SOAP*denovo* (ver. 2.04), Platanus (ver. 1.2.4) and SPAES (ver. 1.2.4) by ORI-GENE (Beijing, China). Multiple Velvet assemblies were constructed using different pairwise combinations of K-mer lengths and expected coverage values (Grewe et al. 2014; Zhu et al. 2014). The resultant mitochondrial contigs were scaffolded into a single chromosome with either the paired-end or mate-pair library using SSPACE 3.0 (Boetzer et al. 2011). Remaining gaps in the *A. mongolicus* mito-genome assembly, which were caused by long mononucleotide repeats (10–20 bp in length), were finished by Sanger sequencing. Features were annotated manually based on the output of NCBI-BLASTN and -BLASTX searches to custom databases. The tRNA genes were annotated using tRNA scan-SE (http://lowelab.ucsc.edu/tRNAscan-SE/) (Lowe and Eddy 1997).

The *A. mongolicus* mito-genome was assembled into a single, circular-mapping molecule of 475,396 bp with 42.75% GC content, which is comparable to the mtDNA sequences of other Leguminosae. The mito-genome contained a total of 127 genes, including 79 protein-coding genes, three rRNA genes inferred to have been present in the ancestral flowering plant mito-genome, and 45 tRNA genes. Among the 79 protein-coding genes, 28 were novel genes, 45 were genes with known functions and six were known orf genes. The tRNA genes encoded 16 intact tRNAs of mitochondrial origin, and 29 intact and potentially functional chloroplast-derived tRNAs.

To examine the phylogenetic evolution of the *A. mongolicus* mitochondria, a neighbor-joining analysis was applied for 18 plant mito-genomes (including *A. mongolicus*) based on the translated amino acid sequences of eight common protein-coding genes (*cob*, *cox1*, *cox3*, *nad6*, *nad9*, *rps12*, *rps3*, *rps4*) and rooted with the one-celled algae, *Chaetosphaeridium globosum*. The topology of the resulting tree was consistent with the representatives in Angiosperm Phylogeny Group APG III. In the phylogenetic tree, the algae and mosses were sisters of land plants, and gymnosperms *Cycas taitungensis* and *Ginkgo biloba* were sisters of the other Angiospermae. Within the Leguminosae clade, *A. mongolicus* was evolutionarily closest to *Glycine max*, and *Medicago truncatula* was a sister of *A. mongolicus* (Figure 1). Each of these relationships had 100% bootstrap support.

**Figure 1.**
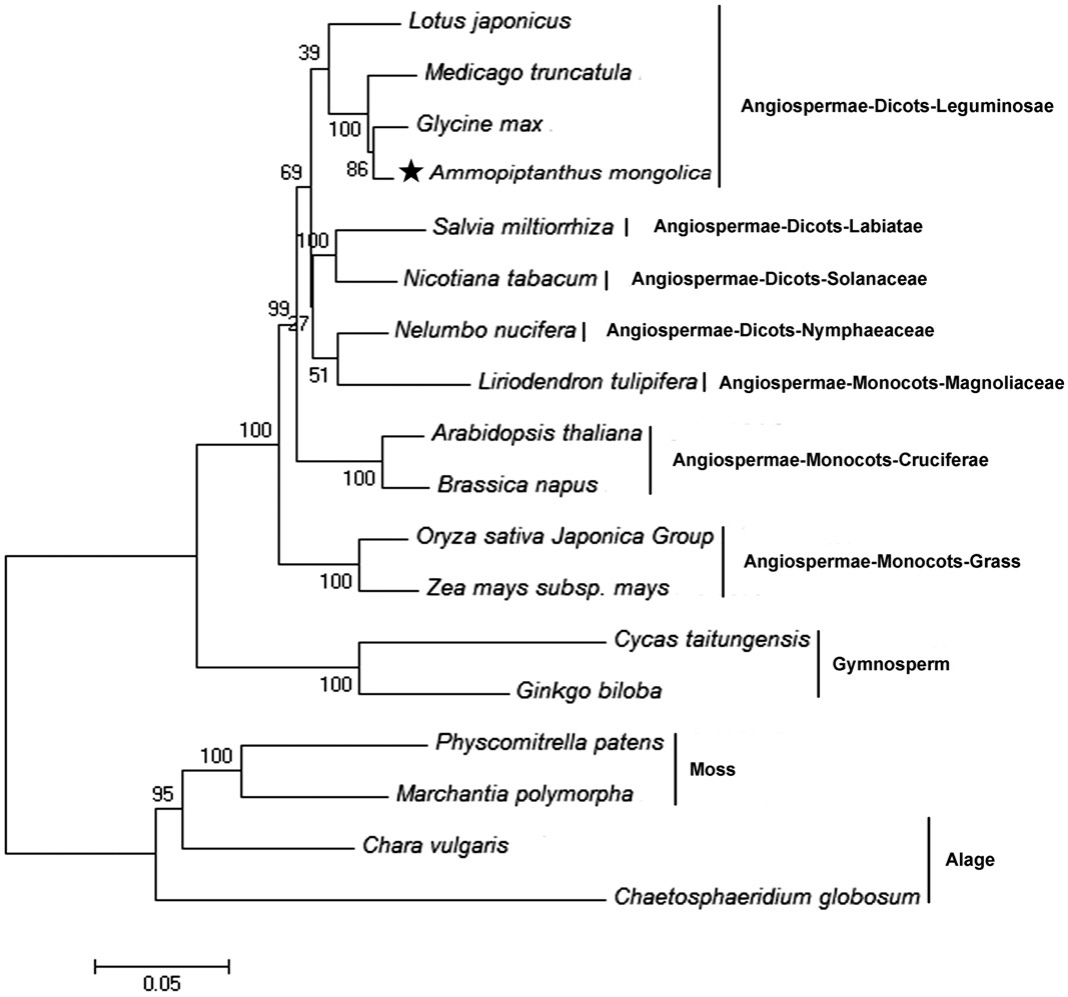
Phylogenetic tree of *Ammopiptanthus mongolicus* and 17 other plant mitochondrial genomes. Neighbour-joining method of 17 amino acid sequences were used, including atp8, atp9, cob, cox1, cox2, cox3, nad1, nad3, nad4L, nad5 and nad6. All the sequences could be currently available in the GenBank database: *Lotus japonicus* (NC_016743), *Medicago truncatula* (NC_029641), *Glycine max* (NC_020455), *Salvia miltiorrhiza* (NC_023209), *Nicotiana tabacum* (NC_006581), *Nelumbo nucifera* (NC_030753), *Liriodendron tulipifera* (NC_021152), *Arabidopsis thaliana* (NC_001284), *Brassica napus* (NC_008285), *Oryza sativa* Japonica Group (NC_011033), *Zea mays* subsp. mays (NC_007982), *Cycas taitungensis* (NC_010303), *Ginkgo biloba* (NC_027976), *Physcomitrella patens* (NC_007945), *Marhantia polynorpha* (NC_001660), *Chara vulgaris* (NC_005255), and *Chaetosphaeridium globosum* (NC_004118); *Chaetosphaeridium globosum* was used as the outgroup. Numbers above each node represent bootstrap values from 1000 replicates. Black star indicate *Ammopiptanthus mongolicus*.
